# Field warming experiments shed light on the wheat yield response to temperature in China

**DOI:** 10.1038/ncomms13530

**Published:** 2016-11-17

**Authors:** Chuang Zhao, Shilong Piao, Yao Huang, Xuhui Wang, Philippe Ciais, Mengtian Huang, Zhenzhong Zeng, Shushi Peng

**Affiliations:** 1 Sino-French Institute for Earth System Science, College of Urban and Environmental Sciences, Peking University, Beijing 100871, China; 2 Key Laboratory of Alpine Ecology and Biodiversity, Institute of Tibetan Plateau Research, Chinese Academy of Sciences, Beijing 100085, China; 3 Chinese Academy of Sciences Center for Excellence in Tibetan Plateau Earth Science, Chinese Academy of Sciences, Beijing 100085, China; 4 State Key Laboratory of Vegetation and Environmental Change, Institute of Botany, Chinese Academy of Sciences, Beijing 100093, China; 5 LSCE, UMR CEA-CNRS, Bat. 709, CE, L'Orme des Merisiers, F-91191 Gif-sur-Yvette, France

## Abstract

Wheat growth is sensitive to temperature, but the effect of future warming on yield is uncertain. Here, focusing on China, we compiled 46 observations of the sensitivity of wheat yield to temperature change (*S*_*Y,T*_, yield change per °C) from field warming experiments and 102 *S*_*Y,T*_ estimates from local process-based and statistical models. The average *S*_*Y,T*_ from field warming experiments, local process-based models and statistical models is −0.7±7.8(±s.d.)% per °C, −5.7±6.5% per °C and 0.4±4.4% per °C, respectively. Moreover, *S*_*Y,T*_ is different across regions and warming experiments indicate positive *S*_*Y,T*_ values in regions where growing-season mean temperature is low, and water supply is not limiting, and negative values elsewhere. Gridded crop model simulations from the Inter-Sectoral Impact Model Intercomparison Project appear to capture the spatial pattern of *S*_*Y,T*_ deduced from warming observations. These results from local manipulative experiments could be used to improve crop models in the future.

## Abstract (web summary)

The degree to which wheat yield in China will respond to future warming remains uncertain. These authors compile data from warming experiments and process-based statistical models and show that warming increases yield only in regions where growing season mean temperature is low and water supply is not limiting.

## Graphical abstract

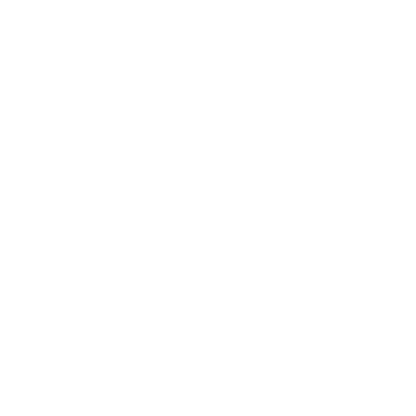

China is the world's largest producer of wheat and life-threatening famine is now a thing of the past. Nevertheless, risks to food security still exist. The increase in population is accompanied by a growth in both the per capita food consumption and the demand for high quality wheat. Most varieties of wheat require both a relatively cool climate in the early growing season and a minimum period of exposure to cold temperature to trigger reproductive development. Future climate warming might thus cause reductions in wheat yield if these conditions are not fulfilled. The recent meta-analysis of future yield projections^1^ used in the Fifth Assessment Report of the Intergovernmental Panel on Climate Change (ref. 2), concluded that without adaptation, a warming of 2 °C should produce an average negative impact on the yield of wheat—although in this analysis, some regions were found to benefit from improving yield with climate change. Documenting the sensitivity of wheat yield to temperature change in different agricultural regions is thus critical to reduce uncertainties on the risks of future yield loss in response to warming. Not few studies investigated regional temperature sensitivities (*S*_*Y,T*_) of wheat yield in China^3^,^4^,^5^,^6^. But their results are not consistent with each other, making it difficult to infer a clear way forward for future adaptation.

Among several approaches to estimate *S*_*Y,T*_, locally calibrated process-based crop models are widely used. These models have equations that describe crop growth and development, typically on a daily time-step. They require extensive input data about cultivar types, climate forcing, management and soil conditions^7^. *S*_*Y,T*_, defined as the partial derivative of simulated wheat yield to temperature, can be diagnosed from these models, for example, by simulating the idealized response of yield to a step-wise or progressive temperature increase^8^,^9^. The simulated values of *S*_*Y,T*_ are model-dependent. Because current crop models are rather complex, differences in *S*_*Y,T*_ between models are difficult to trace back to specific equations and parameters. For instance, a crop model can produce a positive effect of warming on yield through increased carboxylation rates, but warming-induced increases of vapour pressure deficit, photo-respiration or maintenance respiration may negate this positive effect. Statistical modelling is an alternative approach to diagnose *S*_*Y*,*T*_, based on the regression of observed crop yield against climate variables, including temperature^10^,^11^. The value of *S*_*Y*,*T*_ in statistical models depends on the choice of the predictors, and on the assumed empirical relationships (for example, linear versus non-linear response models). The results of statistical models cannot be robustly extrapolated outside the envelope of current-climate to predict future yield changes. Moreover, statistical models have systematic errors, arising from co-linearity between predictor variables^12^. Field warming experiments where temperature is increased artificially over a wheat-cultivated plot, offer an alternative possibility to determine *S*_*Y*,*T*_ at local scale, but the challenge is to scale-up these observations to regional responses of yield to temperature.

Here we compile 46 *S*_*Y,T*_ estimates of the wheat yield response to temperature in China from field warming experiments, and 102 estimates from local process-based and statistical models. All these studies cover major climatic conditions over China's wheat-growing area (Supplementary Fig. 1). Here we compile 46 *S*_*Y,T*_ estimates of the wheat yield response to temperature in China from field warming experiments, and 102 estimates from local process-based and statistical models. All these studies cover major climatic conditions over China's wheat-growing area (Supplementary Fig. 1). Warming experiments show positive *S*_*Y,T*_ values in regions where growing-season mean temperature is low, and water supply is not limiting, and negative values elsewhere. This spatial pattern is captured by global gridded crop models (GGCMs)^13^,^14^ used for the Inter-Sectoral Impact Model Intercomparison Project (ISI-MIP-Phase 1 project)^15^.

## Results

### Average *S*_*Y,T*_ across all studies

**Figure 1:**
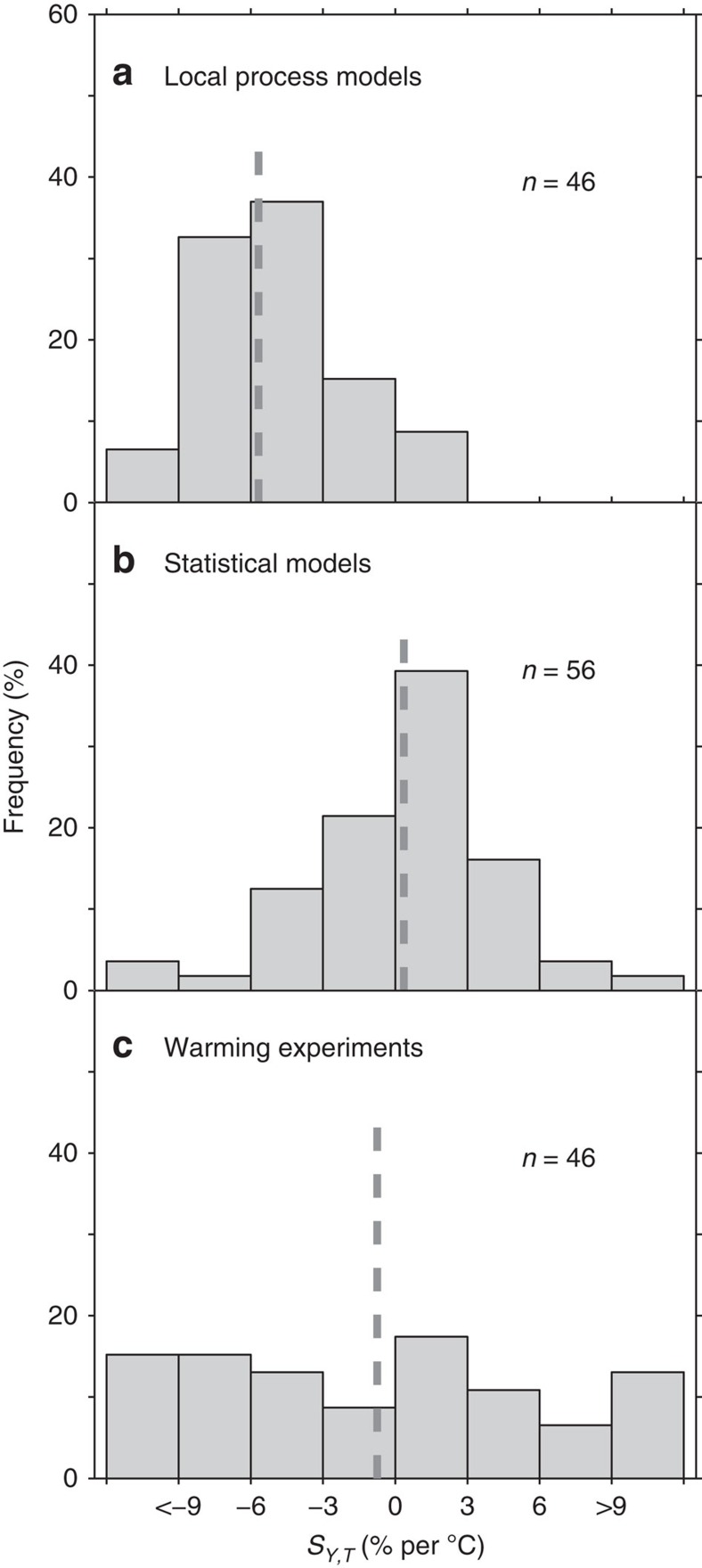
Histograms of *S*_*Y,T*_ derived from different approaches. (**a**) local process models. (**b**) statistical models. (**c**) Field warming experiments. The dotted line refers to the average *S*_*Y,T*_ of each approach and the numbers in the figure give the sample size.

Figure 1 shows that the distributions of temperature sensitivities of wheat yield (*S*_*Y,T*_) differ significantly between warming experiments and the two types of models (local crop models and statistical models). Local crop models give an average negative value of *S*_*Y,T*_ of −5.7% per °C (median *S*_*Y,T*_ is −5.0% per °C) with a large range (s.d.=6.5% per °C; interquartile range (IQR) equals 3.4% per °C). Nevertheless, the results from local crop models are consistent with two recent global meta-analyses^1^,^16^ giving *S*_*Y,T*_ of −4.9% and −3.3±0.8% per °C. By contrast, the statistical models indicate a temperature sensitivity of wheat yield in China that is not statistically different from zero (0.4±4.4% per °C; median *S*_*Y,T*_=0.8% per °C, IQR=4.1% per °C). The independent observed data from the 46 field warming experiments define an average *S*_*Y,T*_ of −0.7±7.8% per °C (median *S*_*Y,T*_=−0.9% per °C with a IQR of 10.8% per °C).

### Regional patterns of *S*_*Y,T*_

**Figure 2:**
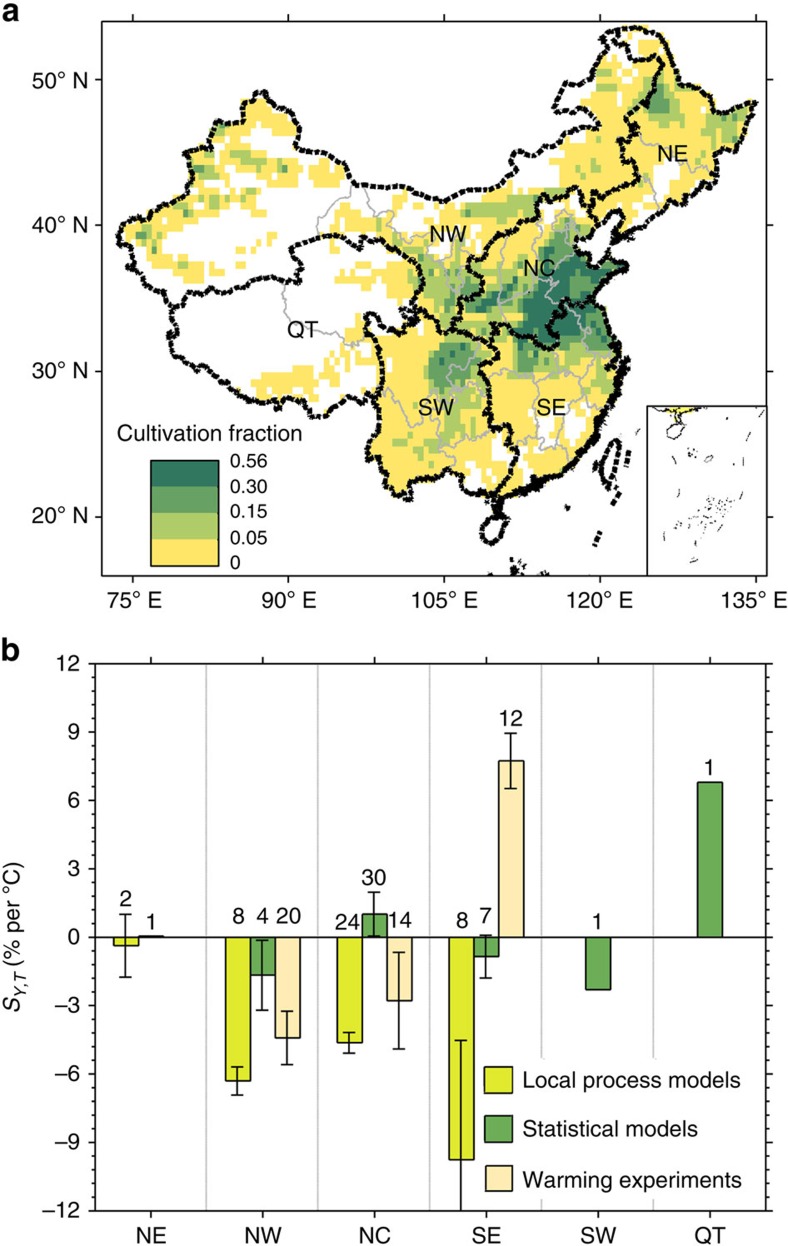
Spatial patterns of wheat cultivation fraction and *S*_*Y,T*_ in China. (**a**) Wheat cultivation fraction of six production zones in China. NE, NW, NC, SE, SW, QT represent Northeast China, Northwest China, North China, Southeast China, Southwest China and Qinghai-Tibet, respectively. (**b**) Regional differences of *S*_*Y,T*_ for different approaches (mean±s.e.m.). The number of observations used in the analysis is shown above each bar. Map was created using Matlab R2014b.

Giving the mean of *S*_*Y,T*_ across a large country like China may mask regionally different values, reflecting diverse climatic conditions and stress factors of wheat-growing areas (Supplementary Table 1). Figure 2 shows the results of regional *S*_*Y,T*_ estimates for the three largest wheat-growing regions, Northwest China (NW), North China (NC) and Southeast China (SW), which altogether comprise 80% of the wheat-cultivated area (90% of the production). Warming experiments and models produce different regional mean *S*_*Y,T*_ values. Namely, the local crop models indicate large negative *S*_*Y,T*_ values in Northwest China, North China and Southeast China (−6.3, −4.6 and −9.8% per °C respectively) but small negative values in Northeast China (−0.4% per °C). Statistical models show positive *S*_*Y,T*_ in North China (1.0% per °C; not statistically significant) and negative values in Northwest and Southeast China (−1.7% and −0.9% per °C; non-significant). The warming experiment results suggest a positive *S*_*Y,T*_ of 7.7% per °C in Southeast China, opposite in sign to the models, and negative *S*_*Y,T*_ of −2.8% per °C and −4.4% per °C in North and Northwest China respectively. In short, the three approaches consistently find a negative impact of warming on yields in Northwest China, yet with different values. While the warming experiments suggest a large positive effect of temperature on yield in Southeast China, models give negative *S*_*Y,T*_ values in this region, that is, a more pessimistic anticipation of the impacts of future warming.

### Relationships between *S*_*Y,T*_ and background climate variables

**Figure 3:**
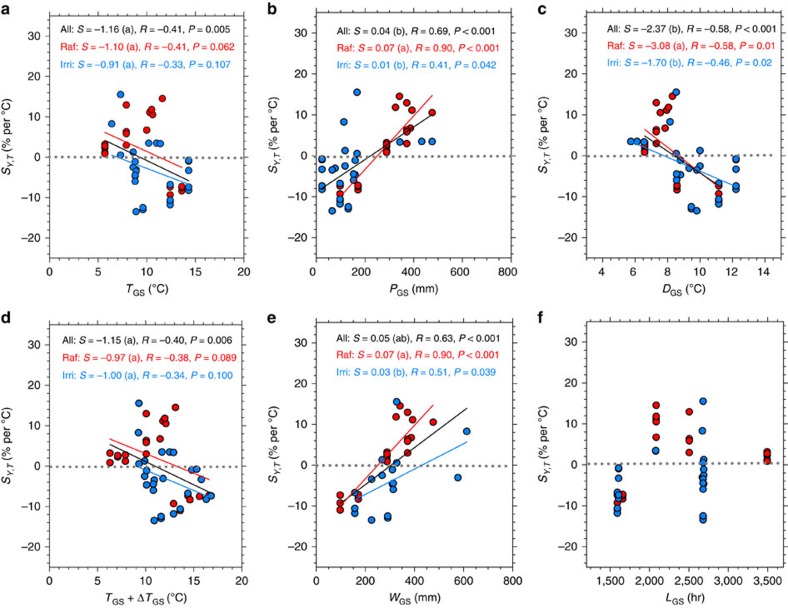
Relationships between *S*_*Y,T*_ and background climate variables for the field warming experiments. (**a**) growing-season temperature (*T*_GS_). (**b**) Growing-season precipitation (*P*_GS_). (**c**) Growing-season diurnal temperature range (*D*_GS_). (**d**) Growing-season increased temperature (*T*_GS_+Δ*T*). (**e**) Growing-season water supply (*W*_GS_; precipitation+irrigation). (**f**) Growing-season daylight hours (*L*_GS_). The data points are grouped into three categories: all included (All; black), rainfed (Raf; red) and irrigated (Irri; blue). *S* represents the slope of the regression line. The same letter (for example, a) in the bracket indicates no significant differences (*P*>0.05) between those categories.

To gain more insights into how regional variations of *S*_*Y,T*_ relate to climate conditions, we performed a linear regression of *S*_*Y,T*_ observations from the warming experiments against growing-season mean temperature (*T*_GS_), water supply (*W*_GS_, defined as the sum of precipitation (*P*_GS_) and irrigation (*I*_GS_)), daylight hours (*L*_GS_) and diurnal temperature range (*D*_GS_; Fig. 3). This regression analysis shows that *S*_*Y,T*_ is negatively correlated with temperature (*R*=−0.41, *P=*0.005), with a 1.2% decrease of *S*_*Y,T*_ across a 1 °C spatial gradient of *T*_GS_. We added the specific warming applied at each site (Δ*T*_GS_) to *T*_GS_ in order to account for the fact that the sites data used in the regression did not experience normal *T*_GS_ conditions, and verified that the relationship remains unchanged (*R*=−0.40, *P*=0.006). We also separated *S*_*Y,T*_ observations into rainfed and irrigated sites (Supplementary Table 2), and found that the relationship with *T*_GS_ or *T*_GS_+Δ*T*_GS_ was still marginally robust and that the regression coefficients did not change (*P*>0.05). Neither local crop models nor statistical models present such a dependency of *S*_*Y,T*_ on *T*_GS_ or *T*_GS_+Δ*T*_GS_ (Supplementary Figs 2 and 3). The linear fit of *S*_*Y,T*_ against *T*_GS_ crosses zero at *T*_GS_=9.3 °C (bootstrapped 90% confidence interval of 7.7–10.8 °C). This implies that in regions where *T*_GS_>9.3 °C, the wheat yield response to warming is negatively correlated with growing-season temperature. Extrapolating space for time leads us to speculate that in regions where *T*_GS_ may surpass this threshold in the future, yield loss might occur in response to rising temperature. At present, Northeast and Northwest China fall into this category of *T*_GS_>9.3 °C; spring wheat cultivation is widespread in those two regions and late spring/summer temperatures can be very hot (Supplementary Fig. 4). The negative *S*_*Y,T*_ values extrapolated from field warming observations over these regions may be related to plant exposure to heat-stress during the grain-filling phase^17^. As of today, however, heat-stress is rarely observed for winter wheat in North and part of Southeast China because harvest occurs in late-May/early-June when seasonal temperature has not yet reached its maximum.

The regression results in Fig. 3 also show that *S*_*Y,T*_ is positively correlated with growing-season water supply (*W*_GS_). This correlation is in fact stronger than with *T*_GS_ (*R*=0.63, *P*<0.001). Without consideration of irrigation, *S*_*Y,T*_ is also positively correlated with *P*_GS_ (*R*=0.69, *P*<0.001). Performing the regression separately with rainfed and irrigated sites does not qualitively change the positive correlation with *P*_GS_. In the local crop models and the statistical models, we did not find a significant relationship between *S*_*Y,T*_ and *P*_GS_ (Supplementary Figs 2 and 5). The positive correlation between *S*_*Y,T*_ and water supply (*W*_GS_) corresponds to an increase of *S*_*Y,T*_ of 4.5% for a positive spatial gradient of 100 mm in *W*_GS_. In rather dry climates below a threshold *W*_GS_ of 305 mm (90% confidence interval: 269–350 mm) *S*_*Y,T*_ crosses zero and becomes negative, but in wetter climates, *S*_*Y,T*_ is always positively related to *W*_GS_. The negative sensitivity where *W*_GS_<305 mm could be from warmer temperature increasing plant transpiration^18^ and accelerating soil moisture depletion, possibly resulting in stress during the late growing season. The positive sensitivity where *W*_GS_>305 mm suggests that above this limit, soil water is sufficient to sustain warming-induced enhanced transpiration, facilitating the recycling and utilization of nutrients and enhancing plant growth and final yield formation.

The regression analysis (Fig. 3c,f) also shows that the association between *S*_*Y,T*_ and *L*_GS_ is insignificant (*P*>0.05), but that *S*_*Y,T*_ is significantly and negatively correlated with *D*_GS_ (*P*<0.05) for both rainfed and irrigated sites. Considering that there is a significant co-variation (co-linearity in the regression) between *D*_GS_ and *T*_GS_ (*R*=0.70, *P*<0.001; Supplementary Fig. 6), the specific dependency of *S*_*Y,T*_ on *D*_GS_ was further tested by making a multi-linear regression with *S*_*Y,T*_ as the response variable and *T*_GS_, *W*_GS_ and *D*_GS_ as predictor variables. The multiple regressions for rainfed (equation (1)) and irrigated conditions (equation (2)) are:


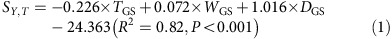


and


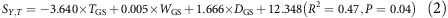


**Figure 4:**
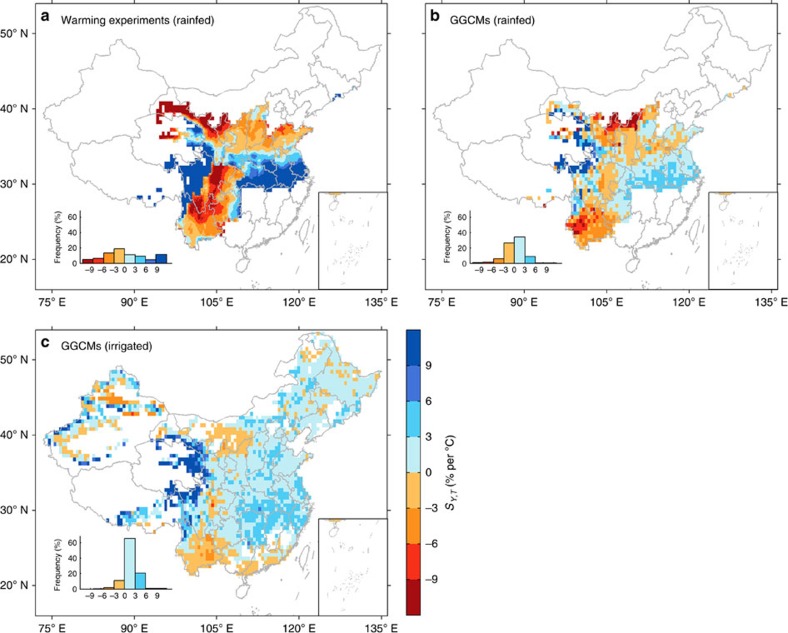
Spatial distribution of *S*_*Y,T*_ in currently rainfed and irrigated wheat-growing areas in China. (**a**) Spatial distribution of *S*_*Y,T*_ extrapolated from field rainfed warming experiments using equation (1), a multiple regression between dependent variable *S*_*Y,T*_ and *T*_GS_, *P*_GS_ and *D*_GS_ averaged from 1981 to 2010. (**b**) Spatial distribution of median *S*_*Y,T*_ diagnosed from rainfed simulations from GGCMs of the ISI-MIP-Phase 1 intercomparison project using equation (3) (see the ‘Methods' section). (**c**) Same as **b** but under fully irrigated scenario. The frequency distribution of *S*_*Y,T*_ weighted by corresponding cultivated wheat area is shown in the histogram inset of each panel. Maps were created using Matlab R2014b.

We found that the regression coefficients of *D*_GS_ are both positive when including *T*_GS_ and *W*_GS_ as predictors. The mechanisms behind this positive response of *S*_*Y,T*_ to *D*_GS_ need to be better understood, but in regions with higher *D*_GS_ and colder nighttime temperatures, warming of nighttime temperature might be more beneficial to wheat growth due to the reduction in frost occurrence^19^,^20^,^21^. We also analysed the relationships between *S*_*Y,T*_ with *D*_GS_ and *L*_GS_ for local process models and statistical models (Supplementary Fig. 2) but found neither of the modelling approaches present the dependency of *S*_*Y,T*_ on *D*_GS_ or *L*_GS_ (*P*>0.05). By utilizing equation (1) with the three predictor variables of *T*_GS_, *W*_GS_ and *D*_GS_, we mapped the spatial distribution of rainfed *S*_*Y,T*_ based on gridded climate data (Fig. 4a). The rainfed based *S*_*Y,T*_ has a spatial pattern similar to the one of *S*_*Y,T*_ derived from all the sites (Supplementary Fig. 7).

### The performance of global gridded crop models

The results from the local crop models do not present the same spatial (temperature or precipitation phase space) distribution of *S*_*Y,T*_ as field warming experiments shown in Fig. 2b. However, one could argue that differences between models contribute to this disagreement. Crop models differ in their structure, complexity and the values of their parameters^22^. Local crop models have been calibrated and tested only for a small region (Supplementary Table 3), and systematic errors arise in extrapolating their results to *S*_*Y,T*_ outside their range of calibration. This is why we also analysed gridded simulations of wheat yield generated by GGCMs^13^,^14^ using the protocol of the ISI-MIP-1 project^15^. GGCMs simulated yield from climate fields (temperature, precipitation and solar radiation from 1971 to 2005), holding constant all other non-climate factors (see the ‘Methods' section) and considered both rainfed and fully irrigated wheat. The spatial distribution of *S*_*Y,T*_ across China diagnosed from a multiple regression between GGCM-simulated wheat yield and climate is presented in Fig. 4b. It can be seen that the results from the GGCMs are more consistent with the field warming experiments regression (equation (1)) than with the local crop model results (Fig. 2b). For instance in Southeast China, the GGCMs' results agree with the warming experiments (Fig. 4a,b) on the positive sign of *S*_*Y,T*_.

The spatial distribution of *S*_*Y,T*_ from the GGCMs simulations under the fully irrigated simulations is also presented in Fig. 4c. Assuming irrigation everywhere, the spatial pattern of *S*_*Y,T*_ across China does not change qualitatively compared with the one of rainfed *S*_*Y,T*_ but is less contrasted (Fig. 4b,c). More importantly, positive *S*_*Y,T*_ are found in North China instead of negative *S*_*Y,T*_ in the rainfed scenario. This is consistent with the results of the warming experiments with fully irrigation in this region published by ref. 23.

## Discussion

Even though only 46 wheat warming experiments were available to this study, these observations are representative of regional and large-scale gradients of the response of wheat yield to temperature in China. Despite their local nature, these observations seem to be useable (after extrapolation with regressions) and give promising support to the gridded crop models. Most of the uncertainty arising from the use of different warming experiments in this study is probably related to differences in experimental methods and imperfectly documented climate conditions from each site^24^,^25^. For example, the use of greenhouses and closed chambers to control higher temperatures has been criticized because it blocks the circulation of air above the plants, and alters light and wind. In the data set compiled here, 34 out of 46 experiments (Supplementary Table 2) used infrared heaters, a method less disruptive than closed chambers^25^. In addition, the magnitude of warming applied at each site might also affect *S*_*Y,T*_, given possible non-linear temperature responses of yield^26^. Yet, we detected no evidence for non-linear effects in our data set (*t*=0.4, *P*=0.7; Supplementary Fig. 8). Last, in addition to local background climate conditions accounted for in equation (1), management conditions at each site could additionally modify the value of *S*_*Y,T*_. We could not test for a complete set of management parameters, but found no significant association between *S*_*Y,T*_ and nitrogen fertilization (*t*=1.6, *P*=0.13) reported at each warming experiment (136−285 kg ha^−1^).

Unlike several warming experiments in US and Europe where sufficient water was applied to determine the direct temperature effects on wheat yield^27^,^28^,^29^, 90% of the warming experiments from China used in this study were rainfed or had limited irrigation (Supplementary Table 2). The temperature sensitivity determined from these data is thus an ‘apparent sensitivity' to temperature, which includes both direct and indirect warming effects, the latter from increased water pressure deficit and higher evaporative demand^17^,^30^. At face value, keeping sites irrigated during experimental warming also mask drought stress (Fig. 4b,c)^31^,^32^. However, quantifying a partial compensation of warming effects by irrigation would need verification in the field, for instance with warming experiments for different irrigation treatments. We also acknowledge the fact that real world experiments with warming and irrigation include atmospheric feedbacks, for example, local evaporative cooling and moistening of the boundary layer, whereas these atmopsheric feedbacks cannot be fully captured in offline crop model simulations.

Field warming experiments show that warmer temperatures do not necessarily lead to a reduction in wheat yield in China. The observed positive yield response to warming for winter wheat in Southeast China and fully irrigated regions of North China might relate to the relatively cool growing-season temperature or non-limiting water supply in these regions (Figs 2 and 3). In addition, warming-induced changes of the growth duration might be another explanation for the positive *S*_*Y,T*_ synthesized from the warming experiment observations. Although artificial warming shortens the overall length of the growth period, it actually extends the active growth period (the growing season without the wintering period)^21^,^23^. A lengthened active growth period enables wheat to extend its grain-filling period and to form yield, which potentially explains the positive warming benefits on the yield of winter wheat.

It should be noted that the primary purpose of this study is not to evaluate models, rather it is to synthesize from different approaches the responses of wheat yield to temperature changes in China and their relationship with background climate. There are several limitations in the comparison of different approaches. First, different approaches have different management and presumably different definitions of growing season, making a rigorous comparison difficult. Second, simulation results are usually averaged over several years or decades, while field warming experiments only reflect the response of yield to temperature during a few years. Thus, background climate likely has more effect in estimates of *S*_*Y,T*_ from annual values (experiments) than from multi-year averages (models; Supplementary Figs 2 and 3). To reduce these sources of systematic errors, robust year-to-year comparisons between modelled and field-measured sensitivities should be performed in future studies.

In summary, this study attempts to assess the temperature sensitivity of wheat yield in China based on three distinct approaches. We found that the two approaches based on field-scale crop models and on statistical models do not show the experimentally observed regional patterns. The warming experiments also suggest the effects of background climate on the temperature sensitivity of wheat yield. The recent gridded crop model ensemble from ISI-MIP1 is however in agreement with the warming experiment data, which gives support to the use of GGCMs for climate impact assessments. Considerable fundamental research on crop physiological response is needed before we will be able to accurately predict how climate change will affect crop yield in China, but our results emphasize that warming-induced yield change is likely to vary across the country and will not always be negative. These findings provide a new perspective on the heterogeneity of the risks to food security, and highlight the importance of developing adaptation options tailored to different regions.

## Methods

### Data sets

We focused on three separate approaches to assess the response of wheat yield to climate change in China: process-based crop models (two types: local agronomical models and generic crop models used for global applications—GGCMs), statistical models and field warming experiments. To integrate results derived from different studies, we used a common measure of temperature sensitivity of wheat yield (*S*_*Y,T*_, yield % change per °C). A literature search was performed on wheat crop yield in China through Web of Science, Google Scholar and China National Knowledge Infrastructure (CNKI; http://www.cnki.net). We considered all peer-reviewed studies published between January 1990 and February 2014 from which *S*_*Y,T*_ values could be calculated. For local process-based models, *S*_*Y,T*_ is usually derived from the difference between a simulation with an arbitrarily increased temperature (for example, +2 °C) and a reference scenario. Field warming experiments employ direct warming treatments (for example, infrared heaters). The experiments are restricted to field scales, and no laboratory or controlled condition experiments are included. For the above two approaches *S*_*Y,T*_ is thus calculated as:


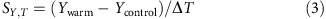


where *Y*_warm_ and *Y*_control_ are the yield from the warmed and control treatment respectively, and Δ*T* is the temperature difference between the warmed and control treatment. The simple regression approach has been widely used by the climate change community^2^,^33^,^34^. Moreover, if the baseline temperature is considered as another fundamental variable and *S*_*Y,T*_ should be calculated from the multiple regression model: Δ*Y*=*S*_*Y,T*_ × Δ*T*+*B* × *T*_base_+*C*. Δ*Y, T*_base_, *B* and *C* represent the yield change, baseline temperature, regression coefficient and intercept term, respectively. However, we then face a practical difficulty because there are no studies that have more than one treatment for both *T*_base_ and Δ*T* to carry out the multiple regressions. For statistical models, *S*_*Y,T*_ is directly extracted from studies that applied multiple regression analysis relating observed wheat yield to independent climate variables. Within an individual study, different amounts of artificial warming, years, cultivars, nutrient and management treatments were considered to be independent, as in previous meta-analyses^35^,^36^,^37^.

To avoid short-term noise and remove the uncertainty from the duration of the applied warming, we focused on the sensitivity of wheat yield to the temperature during the total wheat-growing season (from sowing to maturity). Studies focusing on yield response to short-term temperature change (for example, daytime, nighttime, a particular season or growth period) were discarded in our analysis. Using this criterion, we selected a total of 148 *S*_*Y,T*_ samples in this study. Site descriptions (latitude, longitude, growing-season temperature (*T*_GS_), growing-season precipitation (*P*_GS_) and so on) for each study are given in the Supplementary Tables 2–4. *T*_GS,_ *P*_GS_ and growing-season diurnal temperature range (*D*_GS_; the difference between daytime and nighttime temperature) in the tables are from 0.1° monthly gridded data from Chinese Academy of Sciences of China Meteorological Forcing Dataset (CMFD)^38^ since lots of publications did not report local climate conditions. For regional-scale studies, CMFD climatic data are weighted according to the spatial distribution of wheat cultivation area with 0.5° spatial resolution^39^. For the studies that reported *T*_GS_ and *P*_GS_, we found *T*_GS_ and *P*_GS_ from the CMFD fit well with the corresponding observation data provided in the papers (Supplementary Fig. 9). Note that if two different sites (for example, ref. 2 in Supplementary Table 2) are located in the same 0.1° grid cell of the high resolution climate data set from ref. 38, they are assigned the same *T*_GS_, *P*_GS_ and *D*_GS_, which is one limitation in our analyses. The estimated growing-season length for wheat is from the Chinese Agricultural Phenology Atlas^40^.

### Global gridded crop models

We also applied the output from six GGCMs (EPIC, GEPIC, LPJ-GUESS, LPJml, pDSSAT and pEGASUS) used for ISI-MIP-Phase-1 over the period of 1971–2005 (now available at isi-mip.org). Detailed descriptions of the six GGCMs are provided by ref. 14. All the models were forced with climate reconstruction (temperature, precipitation and solar radiation) based on five Global Climate Models derived from the Coupled Model Intercomparison Project Phase 5 (CMIP5), namely: GFDL-ESM2M, HadGEM2-ES, IPSL-CM5A-LR, MIROC-ESM-CHEM and NorESM1-M.

The simulations were conducted with constant CO_2_, farm technology and nutrition conditions. Rainfed and fully irrigated scenario were divided. Regression analysis was conducted using time series of wheat yield and climatic variables:


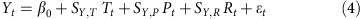


where *Y*_*t*_, *T*_*t*_, *P*_*t*_ and *R*_*t*_ represent growing-season wheat yield, temperature, precipitation and solar radiation in year *t*, respectively. *S*_*Y,T*_, *S*_*Y,P*_ and *S*_*Y,R*_ represent temperature sensitivity, precipitation sensitivity and radiation sensitivity of wheat yield, respectively. *β*_0_ and *ɛ*_*t*_ are the intercept and error term, respectively.

### Data analysis

To investigate whether the distribution of modelling or experimental sites are representative of the country, we made a plot delineating the climate space of wheat-growing areas in China (Supplementary Fig. 1). We found the field-scale experimental and modelling sites we collected well cover the main wheat-growing area in China, though field-scale modelling sites do not cover areas with *P*_GS_ of above 400 mm (12.5% of wheat-growing area in China). To explore the regional variations of *S*_*Y,T*_, the six main production regions are divided with summary information shown in Supplementary Table 1.

To analyse the climate effects on the spatial variations of *S*_*Y,T*_ for field warming experiments, ordinary least squares models were applied to derive the relationships between *S*_*Y,T*_ and the independent climate variables (background growing-season temperature (*T*_GS_), water supply (*W*_GS_; precipitation+irrigation), growing-season diurnal temperature range (*D*_GS_) and daylight hours (*L*_GS_)). *W*_GS_ used in this study only represents water input from rainfall and irrigation rather than available soil moisture to plants. Daylight hours (*L*_GS_) were computed based on the latitude and solar declination at each site^41^. The ordinary least squares models were then used to map the spatial distribution of *S*_*Y,T*_ in observed area where rainfed wheat grows in China^42^ based on gridded CMFD baseline climate data (1981–2010). For local process-based and statistical models, irrigation amount was not reported in the literature, thus we only explored the relationship between *S*_*Y,T*_ and *P*_GS_. It should be noted that for the sites where precipitation is below 100 mm, irrigation was applied to sustain the growth of wheat (Supplementary Table 2). We also analysed this relationship by separating rainfed and irrigated management for local process-based models, but we could not do that for statistical models since the management could not be separated using the descriptions in the literature.

Similarly, for some papers the authors only reported multi-season averages, and did not give site-season results (Supplementary Table 2). Even so, this limitation should not significantly influence our spatial analysis as the interannual variability of baseline temperature at each site is much smaller (<20%) than the spatial gradient of base temperature, which is the main point we focused on. Moreover, for experimental sites (a total of four) that reported a multi-season average, we bootstrapped all different years of baseline temperature and redid the regression to see whether the results still remained robust (Supplementary Fig. 10).

It should also be noted that we did not make separate analyses for winter wheat and spring wheat, since the dominant majority (93%; National Bureau of Statistics of China, 2012; http://www.stats.gov.cn) of wheat grown in China is winter wheat, and we had only three experimental sites having data on spring wheat (occupying 7% of wheat area), making the separate analyses problematic in a statistical sense.

### Data availability

The authors declare that the data supporting the findings of this study are available within the article and its Supplementary Information files.

## Additional information

**How to cite this article:** Zhao, C. *et al*. Field warming experiments shed light on the wheat yield response to temperature in China. *Nat. Commun.* **7,** 13530 doi: 10.1038/ncomms13530 (2016).

**Publisher's note:** Springer Nature remains neutral with regard to jurisdictional claims in published maps and institutional affiliations.

## Supplementary Material

Supplementary Information Supplementary Figures 1-10, Supplementary Tables 1-4 and Supplementary References
