# Supplementary material for: Field warming experiments shed light on the wheat yield response to temperature in China
Source: Nat Commun. 2016 Nov 17;7:13530. doi: 10.1038/ncomms13530 (PMC5118553; doi:10.1038/ncomms13530)
Supplement: Supplementary Information — Supplementary Figures 1-10, Supplementary Tables 1-4 and Supplementary References [file ncomms13530-s1.pdf]

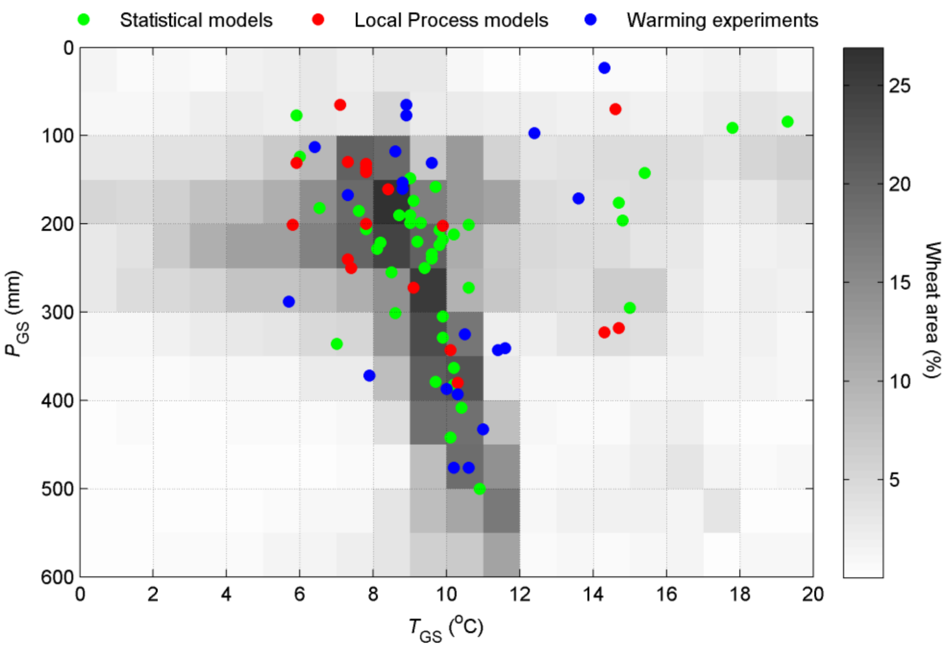

Supplementary Figure 1. The distribution of sites or regions in climate coordinates for three different approaches. The right color bar indicates the fraction of wheat-growing area.

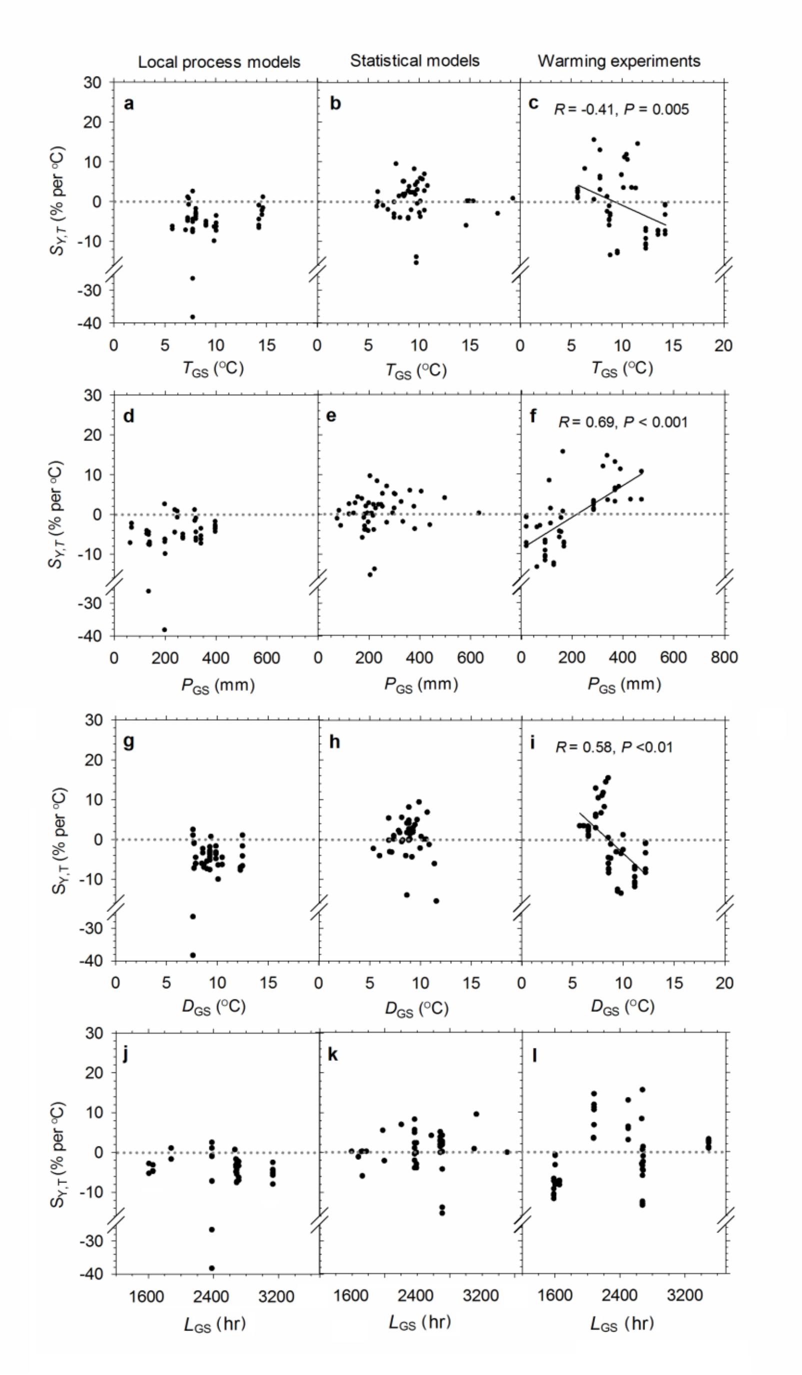

Supplementary Figure 2. Relationships between $S_{Y,T}$ and local climate variables for different approaches. (a-c) growing-season temperature (T_{GS})**. (d-f)** growing-season precipitation (P_{GS}). **(g-i)** the difference between daytime and nighttime temperature (D_{GS}). **(j-l)** growing-season daylight hours (L_{GS}).

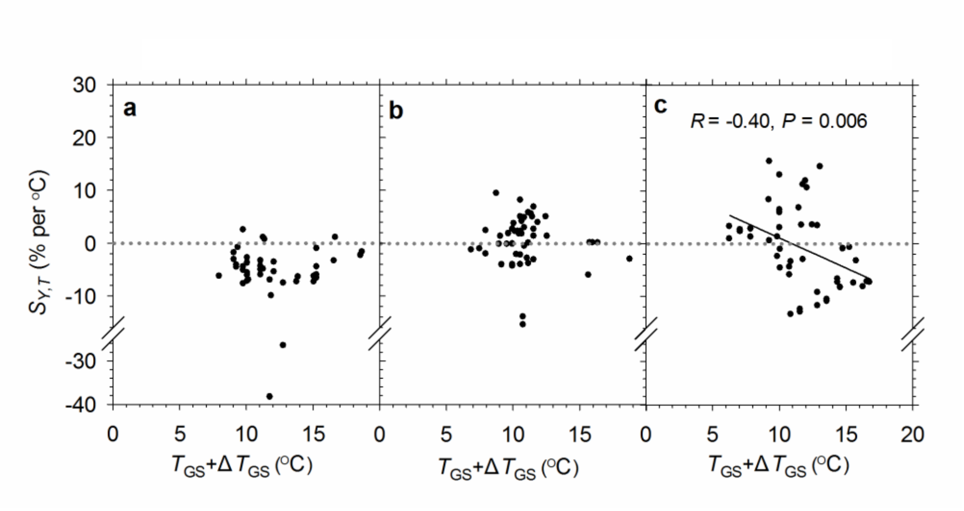

Supplementary Figure 3. Relationships between $S_{Y,T}$ and growing-season increased temperature (T_{GS}+ΔT_{GS}) for different approaches. (a) local process models**. (b)** statistical models**. (c)** field warming experiments.

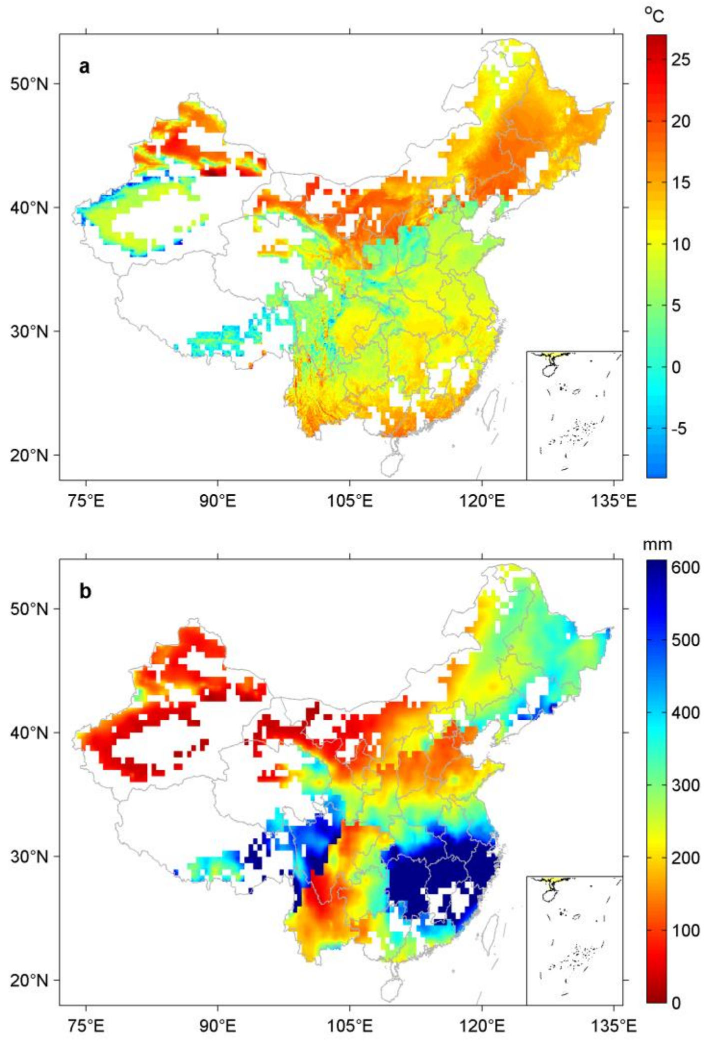

Supplementary Figure 4. **Spatial distribution of growing-season climate variables for wheat in China averaged from 1981-2010**. **(a)** growing-season mean temperature**. (b)** growing-season total precipitation**.** Maps were created using Matlab R2014b.

Editorial note: Springer Nature remains neutral with regard to jurisdictional claims in published maps and institutional affiliations.

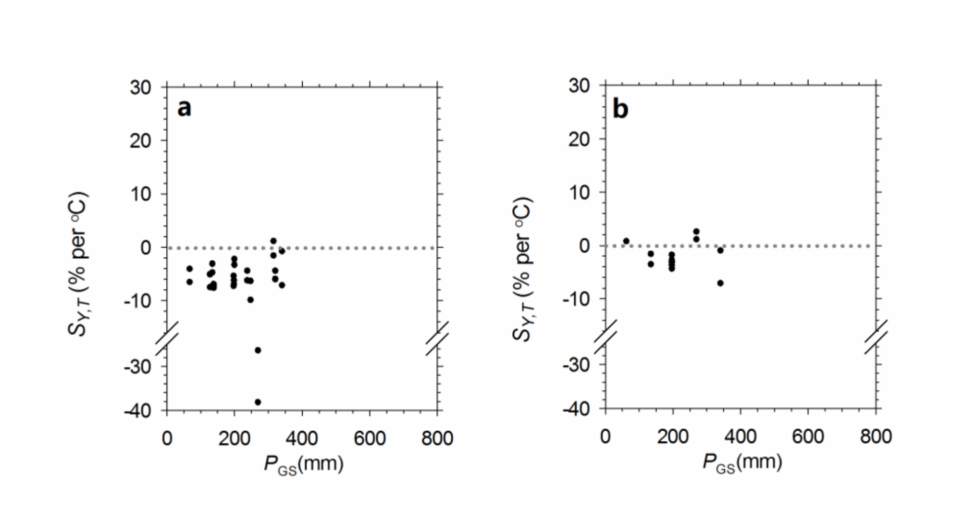

Supplementary Figure 5. Relationships between $S_{Y,T}$ and P_{GS} for local process-based crop models. (a) rainfed. **(b)** irrigated**.**

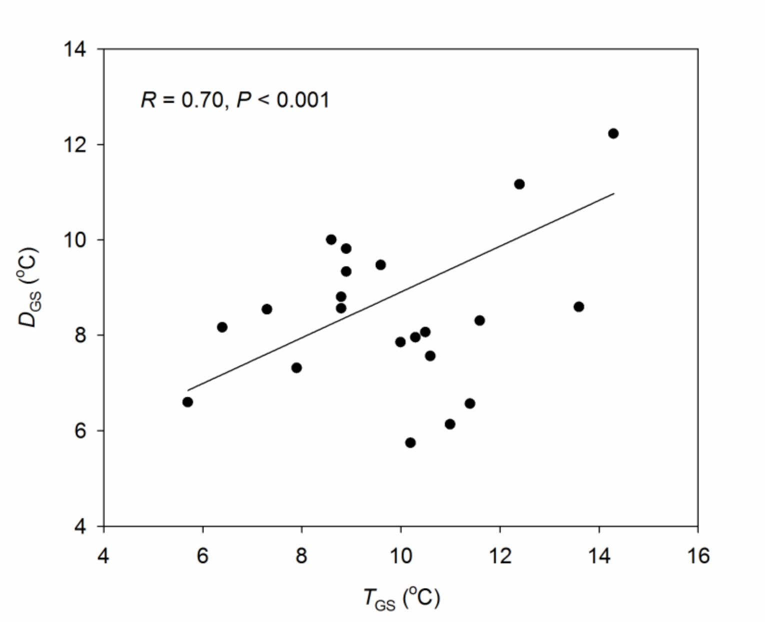

Supplementary Figure 6. Relationship between T_{GS} and D_{GS} for the field warming experiments.

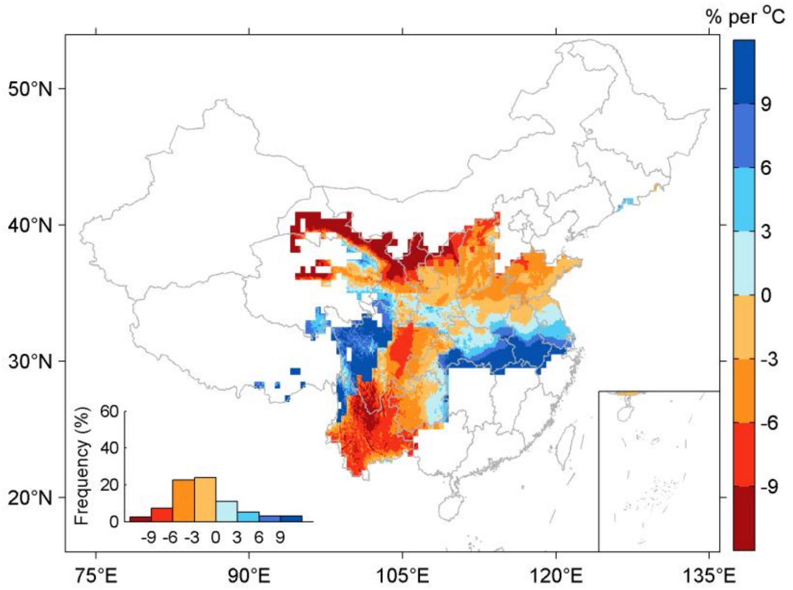

Supplementary Figure 7. Same as Figure 4a in the main text but the spatial pattern of $S_{Y,T}$ was extrapolated by using all the field warming experiments. Map was created using Matlab R2014b.

Editorial note: Springer Nature remains neutral with regard to jurisdictional claims in published maps and institutional affiliations.

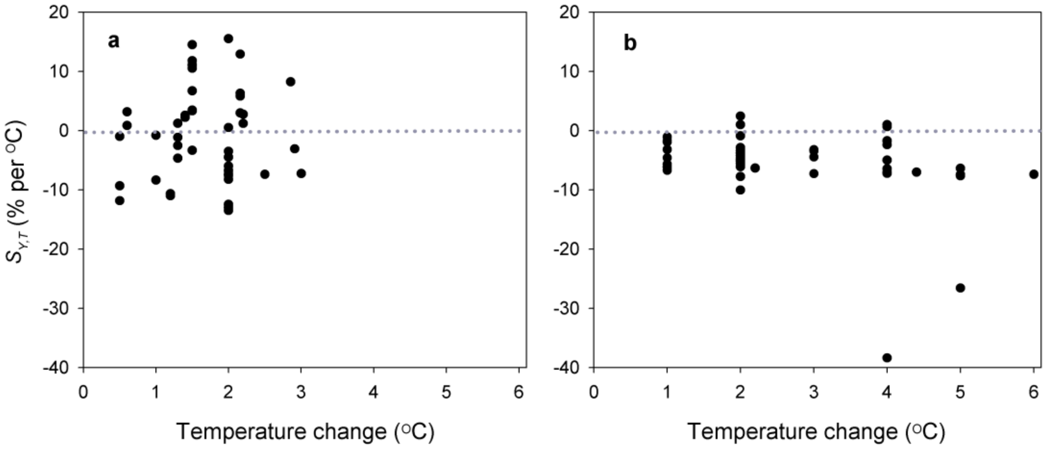

Supplementary Figure 8. ***$S_{Y,T}$* under different magnitudes of warming. (a)** field warming experiments. **(b)** local process-based models.

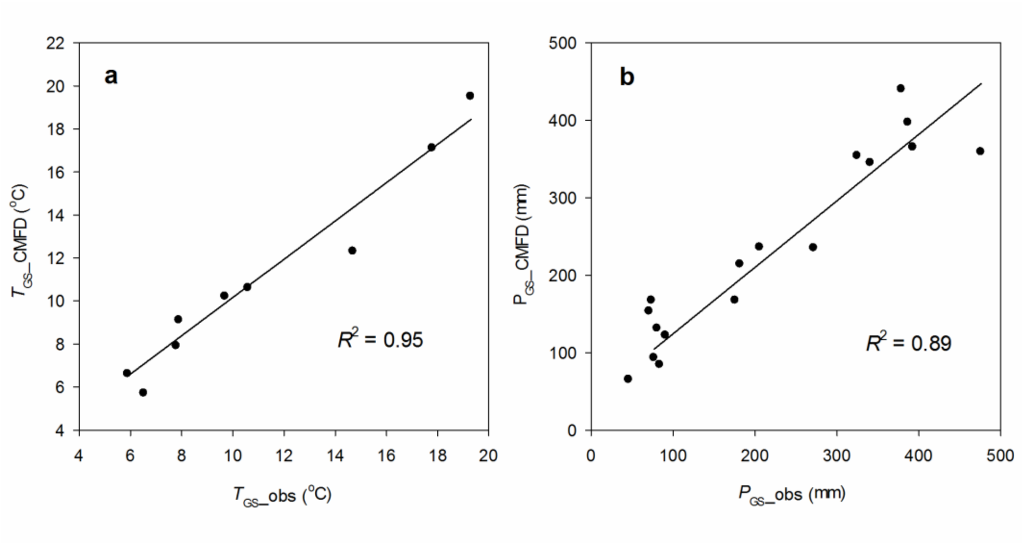

Supplementary Figure 9. **Comparison between China Meteorological Forcing Dataset (CMFD) and the climate data given in the published studies**. (**a**), growing-season temperature (T_{GS}) from CMFD versus the given T_{GS}. (**b**), growing-season precipitation (P_{GS}) for CMFD versus the given P_{GS}.

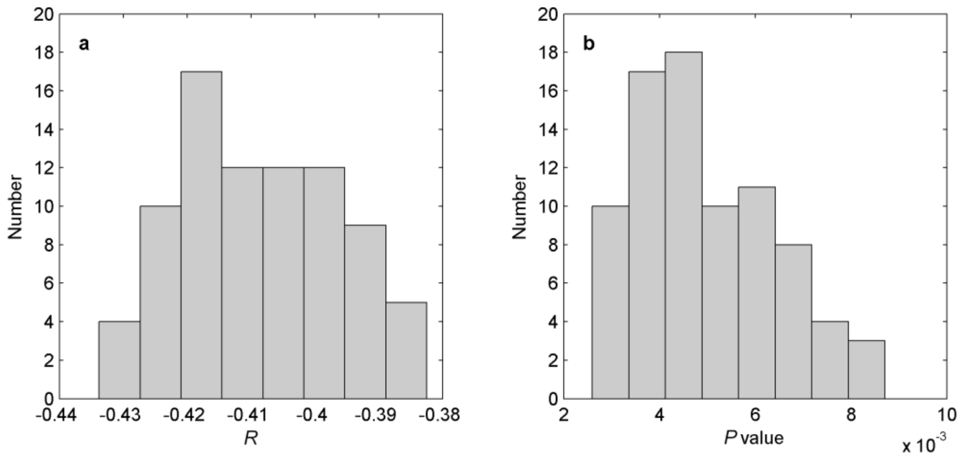

Supplementary Figure 10. Histograms of correlation coefficients and their corresponding p values between $S_{Y,T}$ and T_{GS} by bootstrapping all different years of baseline temperature and redoing the correlation analysis for experimental sites that reported multi-season average. (a) correlation coefficients. **(b)** p values.

**Wheat region**	**Wheat type**	**Yield* (t/ha)**	**Growing season (month)**	T_{GS} **(ºC)**	P_{GS} **(mm)**
NE	Spring wheat	3.4	4-7	15.1	303
NW	Spring wheat	3.9	4-7	15.1	172
	Winter wheat	4.3	10-6	6.3	146
NC	Winter wheat	5.6	10-5	8.1	225
SE	Winter wheat	3.0	11-3,4,5	9.9	423
SW	Winter wheat	4.8	11-4	10.0	199
QT	Spring wheat	4.0	4-8	10.3	256
	Winter wheat	6.5	10-8	2.4	378

Supplementary Table 1. **General information on the six wheat production zones in China.** NE, NW, NC, SE, SW, QT represent Northeast China, Northwest China, North China, Southeast China, Southwest China and Qinghai-Tibet, respectively. T_{GS} and P_{GS} represent growing-season temperature and precipitation, respectively.*The yield data were obtained from China's National Statistical Bureau (2012, http://www.stats.gov.cn)

Wheat type	Site name	Latitude (ºN)	Longitude (ºE)	Measurement period	Warming design	Warming degree(ºC)	Irrigation (mm)*	Nitrogen (kg/ha)	T_{GS} (ºC)	P_{GS} (mm)	D_{GS} (ºC)	Sensitivity (% per ºC)	Ref.
Winter wheat	Nanjing	32.03	118.86	11/2004-05/2005.	Infrared heaters	1.5	0	225	10.3	393	8.0	11.1	1
Winter wheat	Nanjing	32.03	118.86	11/2005-05/2006	Infrared heaters	1.5	0	225	10.6	476	7.6	10.5	1
Winter wheat	Nanjing	32.03	118.86	11/2006-05/2007	Infrared heaters	1.5	0	225	11.6	341	8.3	14.5	1
Winter wheat	Nanjing	32.03	118.86	11/2007-05/2008	Infrared heaters	1.5	0	225	10.0	387	7.9	6.7	1
Winter wheat	Nanjing	32.03	118.86	11/2008-05/2009	Infrared heaters	1.5	0	225	10.5	325	8.1	11.8	1
Winter wheat	Tongwei County	35.22	105.23	09/2006-06/2007<br>09/2007-06/2008	Heating cable	0.6	0	N/A	5.7	288	6.6	3.17	2
Winter wheat	Tongwei County	35.22	105.23	09/2006-06/2007<br>09/2007-06/2008	Heating cable	1.4	0	N/A.	5.7	288	6.6	2.21	2
Winter wheat	Tongwei County	35.22	105.23	09/2006-06/2007<br>09/2007-06/2008	Heating cable	2.2	0	N/A	5.7	288	6.6	1.18	2
Winter wheat	Lulu Mountain	35.29	105.26	09/2006-06/2007<br>09/2007-06/2008	Heating cable	0.6	0	N/A	5.7	288	6.6	0.83	2
Winter wheat	Lulu Mountain	35.29	105.26	09/2006-06/2007<br>09/2007-06/2008	Heating cable	1.4	0	N/A	5.7	288	6.6	2.57	2
Winter wheat	Lulu Mountain	35.29	105.26	09/2006-06/2007<br>09/2007-06/2008	Heating cable	2.2	0	N/A	5.7	288	6.6	2.73	2
Spring wheat	Guyuan	36.03	106.46	03/2001-06/2001<br>03/2004-06/2004	Greenhouse	0.5	0	N/A	12.4	97	11.2	-9.33	3
Spring wheat	Guyuan	36.03	106.46	03/2001-06/2001<br>03/2004-06/2004	Greenhouse	1.2	0	N/A	12.4	97	11.2	-11.03	3
Spring wheat	Guyuan	36.03	106.46	03/2001-06/2001<br>03/2004-06/2004	Greenhouse	2.0	0	N/A	12.4	97	11.2	-7.4	3

Spring wheat	Guyuan	36.03	106.46	03/2001-06/2001<br>03/2004-06/2004	Greenhouse	0.5	60	N/A	12.4	97	11.2	-11.84	3
Spring wheat	Guyuan	36.03	106.46	03/2001-06/2001<br>03/2004-06/2004	Greenhouse	1.2	60	N/A	12.4	97	11.2	-10.65	3
Spring wheat	Guyuan	36.03	106.46	03/2001-06/2001<br>03/2004-06/2004	Greenhouse	2.0	60	N/A	12.4	97	11.2	-6.78	3
Winter wheat	Dingxing	39.13	115.66	10/2008-05/2009	Infrared heaters	2.9	500	210	8.9	77	9.3	-3.1	4
Winter wheat	Dingxing	39.13	115.66	10/2009-05/2010	Infrared heaters	2.9	500	210	6.4	113	8.2	8.25	4
Spring wheat	Xidatan	38.80	106.30	03/2011-06/2011	Infrared heaters	0.5	N/A	136	14.3	23	12.2	-1.0	5
Spring wheat	Xidatan	38.80	106.30	03/2011-06/2011	Infrared heaters	1.0	N/A	136	14.3	23	12.2	-0.8	5
Spring wheat	Xidatan	38.80	106.30	03/2011-06/2011	Infrared heaters	1.5	N/A	136	14.3	23	12.2	-3.33	5
Spring wheat	Xidatan	38.80	106.30	03/2011-06/2011	Infrared heaters	2.0	N/A	136	14.3	23	12.2	-8.25	5
Spring wheat	Xidatan	38.80	106.30	03/2011-06/2011	Infrared heaters	2.5	N/A	136	14.3	23	12.2	-7.4	5
Winter wheat	Shanghai	31.21	121.13	12/2007-05/2008	Infrared heaters	1.5	N/A	N/A	11.0	433	6.1	3.46	6
Winter wheat	Shanghai	31.21	121.13	12/2008-05/2009	Infrared heaters	1.5	N/A	N/A	11.4	343	6.6	3.33	6
Winter wheat	Shanghai	31.21	121.13	12/2009-05/2010	Infrared heaters	1.5	N/A	N/A	10.2	476	5.7	3.45	6
Winter wheat	Yucheng	36.83	116.57	10/2010-05/2011	Infrared heaters	1.3	150	285	8.6	118	10.0	1.23	7
Winter wheat	Yucheng	36.83	116.57	10/2010-05/2011	Infrared heaters	1.3	150	285	8.6	118	10.0	-2.54	7
Winter wheat	Yucheng	36.83	116.57	10/2011-05/2012	Infrared heaters	1.3	150	285	8.8	160	8.8	-1.15	7
Winter wheat	Yucheng	36.83	116.57	10/2011-05/2012	Infrared heaters	1.3	150	285	8.8	160	8.8	-4.69	7
Winter wheat	Lianyungang	34.55	119.39	10/2009-05/2010	Infrared heaters	2.2	0	0	7.9	372	7.3	12.92	8
Winter wheat	Lianyungang	34.55	119.39	10/2009-05/2010	Infrared heaters	2.2	0	150	7.9	372	7.3	6.31	8
Winter wheat	Lianyungang	34.55	119.39	10/2009-05/2010	Infrared heaters	2.2	0	225	7.9	372	7.3	2.95	8
Winter wheat	Lianyungang	34.55	119.39	10/2009-05/2010	Infrared heaters	2.2	0	300	7.9	372	7.3	5.82	8

Winter wheat	Luancheng	37.88	114.68	10/2008-05/2009	Infrared heaters	2.0	160	240	9.6	131	9.5	-13.0	9
Winter wheat	Luancheng	37.88	114.68	10/2010-05/2011	Infrared heaters	2.0	160	240	8.9	65	9.8	-13.5	9
Winter wheat	Luancheng	37.88	114.68	10/2011-05/2012	Infrared heaters	2.0	160	240	8.8	153	8.6	-6.0	9
Winter wheat	Luancheng	37.88	114.68	10/2008-05/2009	Infrared heaters	2.0	160	0	9.6	131	9.5	-12.5	9
Winter wheat	Luancheng	37.88	114.68	10/2010-05/2011	Infrared heaters	2.0	160	0	8.9	65	9.8	-3.5	9
Winter wheat	Luancheng	37.88	114.68	10/2011-05/2012	Infrared heaters	2.0	160	0	8.8	153	8.6	-4.5	9
Winter wheat	Luancheng	37.88	114.68	10/2009-05/2010	Infrared heaters	2.0	160	240	7.3	167	8.5	15.5	9
Winter wheat	Luancheng	37.88	114.68	10/2009-05/2010	Infrared heaters	2.0	160	0	7.3	167	8.5	0.5	9
Spring wheat	Dingxi	35.58	104.62	04/2010-07/2010<br>04/2011-07/2011	Infrared heaters	1.0	0	N/A	13.6	171	8.6	-8.37	10
Spring wheat	Dingxi	35.58	104.62	04/2010-07/2010<br>04/2011-07/2011	Infrared heaters	2.0	0	N/A	13.6	171	8.6	-7.55	10
Spring wheat	Dingxi	35.58	104.62	04/2010-07/2010<br>04/2011-07/2011	Infrared heaters	3.0	0	N/A	13.6	171	8.6	-7.27	10

Supplementary Table 2. **The temperature sensitivity of wheat yield and relevant information for field warming experiments in China.** N/A means that the information is not available in that paper. D_{GS} represent growing-season diurnal temperature range. * No irrigation (zero) indicated that wheat was rainfed. Experiments where additional water than precipitation was supplied to wheat (e.g. ref. 6) was considered as irrigated. In ref. 2, the two sites (Tongwei county and Lulu mountain) were located in the same 0.1 ^{o} grid cell, having the same T_{GS} and P_{GS} in the high resolution gridded climate data used for those two sites. In ref. 2, 3 and 10, authors only reported the average temperature responses for multiple seasons, and T_{GS} and P_{GS} were thus sampled to cover the same period of the observed data. In *ref*. 5, irrigation was applied at seedling, elongating, and flowering stages but the actual amounts applied were not available (N/A). In *ref. 6* an underground system was built to maintain

the water table at 50 cm below soil surface and the actual irrigation amounts were not available, so the data in *ref. 5* and *ref. 6* were not included in Fig. 3d in the main text. In *ref. 10* a rain shelter was used to remove the rainfall when raining and it irrigated a total of 171 mm water during the wheat growth period. This fixed irrigation amount was exactly the average precipitation of the site over the past 30 years (from 1978-2009). Therefore, the site had, in fact, similar water treatment as rainfed crops and the data in *ref. 10* was kept in rainfed category in Fig. 3 in the main text.

Wheat type	Location	Latitude (ºN)	Longitude (ºE)	Simulation period	Crop model used	Calibration	Warming Degree(ºC)	T_{GS} (ºC)	P_{GS} (mm)	D_{GS} (ºC)	Sensitivity (% per ºC)	Management	Note(s)	Ref.
Spring wheat	Mohe	52.97	122.51	1981-2000	CERES-wheat	Yes	2.0	14.7	318	12.5	1.0	rainfed		11
Spring wheat	Mohe	52.97	122.51	1981-2000	CERES-wheat	Yes	4.0	14.7	318	12.5	-1.75	rainfed		11
Winter wheat	Bengbu	32.95	117.38	1980-1989	SUCROS	Yes	1.0	9.1	272	7.6	1.0	irrigated		12
Winter wheat	Bengbu	32.95	117.38	1980-1989	SUCROS	Yes	2.0	9.1	272	7.6	2.45	irrigated		12
Winter wheat	Bengbu	32.95	117.38	1980-1989	SUCROS	Yes	1.0	9.1	272	7.6	-38.4	rainfed		12
Winter wheat	Bengbu	32.95	117.38	1980-1989	SUCROS	Yes	2.0	9.1	272	7.6	-26.6	rainfed		12
Spring wheat	Dingxi	35.47	104.73	2005	APSIM-wheat	Yes	1.0	14.3	323	7.9	-6.2	rainfed	Tillage	13
Spring wheat	Dingxi	35.47	104.73	2005	APSIM-wheat	Yes	1.0	14.3	323	7.9	-6.1	rainfed	No tillage	13
Spring wheat	Dingxi	35.47	104.73	2005	APSIM-wheat	Yes	1.0	14.3	323	7.9	-4.6	rainfed	No tillage +mulch	13
Winter wheat	Hebei Province			2000-2004	WOFOST	Yes	2.0	7.3	130	9.3	-5.25	rainfed		14
Winter wheat	Hebei Province			2000-2004	WOFOST	Yes	4.0	7.3	130	9.3	-7.65	rainfed		14
Winter wheat	Shanxi Province			2000-2004	WOFOST	Yes	2.0	7.3	240	10.5	-4.6	rainfed		14
Winter wheat	Shanxi Province			2000-2004	WOFOST	Yes	4.0	7.3	240	10.5	-6.38	rainfed		14
Winter wheat	Shandong Province			2000-2004	WOFOST	Yes	2.0	7.8	200	9.0	-5.55	rainfed		14
Winter wheat	Shandong Province			2000-2004	WOFOST	Yes	4.0	7.8	200	9.0	-7.45	rainfed		14
Winter wheat	Botou	38.18	116.52	1996-2004	VIP	N/A	5.0	7.8	137	9.9	-3.7	irrigated		15
Winter wheat	Botou	38.18	116.52	1996-2004	VIP	N/A	2.0	7.8	137	9.9	-1.75	irrigated		15
Winter wheat	Botou	38.18	116.52	1996-2004	VIP	N/A	5.0	7.8	137	9.9	-4.92	rainfed		15
Winter wheat	Botou	38.18	116.52	1996-2004	VIP	N/A	2.0	7.8	137	9.9	-3.25	rainfed		15
Winter wheat	Huaiyuan	32.56	117.21	1996-2004	VIP	N/A	5.0	10.1	343	7.7	-7.24	irrigated		15

Winter wheat	Huaiyuan	32.56	117.21	1996-2004	VIP	N/A	2.0	10.1	343	7.7	-1.1	irrigated		15
Winter wheat	Huaiyuan	32.56	117.21	1996-2004	VIP	N/A	5.0	10.1	343	7.7	-7.3	rainfed		15
Winter wheat	Huaiyuan	32.56	117.21	1996-2004	VIP	N/A	2.0	10.1	343	7.7	-0.9	rainfed		15
	China			1986-1990	GLAM-wheat	N/A	1.0				-5.7			16
	China			1986-1990	GLAM-wheat	N/A	2.0				-5.1			16
	China			1986-1990	GLAM-wheat	N/A	3.0				-5.0			16
	China			1986-1990	GLAM-wheat	N/A	4.0				-4.65			16
Spring wheat	Dingxi	35.47	104.73	2002-2005	APSIM-wheat	Yes	1.0	13.9	236	8.5	-6.1	rainfed		17
Winter wheat	Beijing	39.70	116.8	2005-2006	CERES-wheat	Yes	3.0	7.1	65	9.4	0.65	irrigated		18
Winter wheat	Tianshui	34.58	105.75	2000-2004	CERES-wheat	Yes	2.0	7.4	250	10.1	-10.05	rainfed		19
Winter wheat	Tianshui	34.58	105.75	2000-2004	CERES-wheat	Yes	4.0	7.4	250	10.1	-6.48	rainfed		19
Winter wheat	Xianyang	34.40	108.71	2000-2004	CERES-wheat	Yes	2.0	9.9	202	8.6	-3.45	rainfed		19
Winter wheat	Xianyang	34.40	108.71	2000-2004	CERES-wheat	Yes	4.0	9.9	202	8.6	-2.4	rainfed		19
Spring wheat	Yongning	38.25	106.23	2000-2004	CERES-wheat	Yes	2.0	14.6	70	12.5	-6.7	rainfed		19
Spring wheat	Yongning	38.25	106.23	2000-2004	CERES-wheat	Yes	4.0	14.6	70	12.5	-4.23	rainfed		19
Winter wheat	Taigu	37.43	112.58	1999-2001	CERES-wheat	Yes	2.0	8.1	121	12.3	-7.8	rainfed		20
Winter wheat	Taigu	37.43	112.58	1999-2001	CERES-wheat	Yes	4.0	8.1	121	12.3	-7.08	rainfed		20
Winter wheat	Taigu	37.43	112.58	1999-2001	CERES-wheat	Yes	6.0	8.1	121	12.3	-7.4	rainfed		20
Winter wheat	Changwu	35.20	107.80	1950-1999	WEEP	Yes	2.2	5.4	207	8.7	-6.36	rainfed		21
Winter wheat	Changwu	35.20	107.80	1950-1999	WEEP	Yes	4.4	5.4	207	8.7	-7.05	rainfed		21
Winter wheat	North China Plain			1980-2009	APSIM-wheat	Yes	1.0	8.1	199	9.3	-3.2	irrigated	Cultivar 1	22
Winter wheat	North China Plain			1980-2009	APSIM-wheat	Yes	1.0	8.1	199	9.3	-1.92	irrigated	Cultivar 2	22
Winter wheat	North China Plain			1980-2009	APSIM-wheat	Yes	2.0	8.1	199	9.3	-3.84	irrigated	Cultivar 1	22
Winter wheat	North China Plain			1980-2009	APSIM-wheat	Yes	2.0	8.1	199	9.3	-2.88	irrigated	Cultivar 2	22

Winter wheat	North China Plain	1980-2009	APSIM-wheat	Yes	3.0	8.1	199	9.3	-4.48	irrigated	Cultivar 1	22
Winter wheat	North China Plain	1980-2009	APSIM-wheat	Yes	3.0	8.1	199	9.3	-3.41	irrigated	Cultivar 2	22

Supplementary Table 3. **The temperature sensitivity of wheat yield and relevant information from process-based crop model sensitivity simulations for China.** N/A means that the information is not available in that paper.

Wheat type	Location	Latitude (ºN)	Longitude (ºE)	Research time	Warming Degree(ºC)	T_{GS} (ºC)	P_{GS} (mm)	D_{GS} (ºC)	Sensitivity (% per ºC)	Ref.
Spring wheat	Inner Mongolia			1975-2008	1.0	14.8	196	10.3	0.04	23
Spring wheat	Northwest China			1975-2008	1.0	15.4	142	10.6	0.04	23
Spring wheat	Northeast China			1975-2008	1.0	15	295	9.0	0.05	23
Spring wheat	South China			1975-2008	1.0	20.8	635	7.4	0.04	23
Winter wheat	Central China			1975-2008	1.0	8.2	221	8.9	-0.23	23
Winter wheat	Northwest China			1975-2008	1.0	7	336	8.3	-0.17	23
Winter wheat	South China			1975-2008	1.0	10.2	363	6.9	-0.23	23
Winter wheat	Kunshan	31.41	120.95	1990-2009	1.0	10.9	500	6.0	-4.1	24
Winter wheat	Yangzhou	32.41	119.41	1990-2009	1.0	10.1	442	7.2	-3.2	24
Winter wheat	Huaiyin	33.60	119.03	1990-2009	1.0	10.2	382	8.0	1.7	24
Winter wheat	North NCP			1956-1985	2.0	6	124	9.7	4.95	25
Winter wheat	North NCP			1956-1985	3.0	6	124	9.7	4.97	25
Winter wheat	North NCP			1956-1985	4.0	6	124	9.7	4.98	25
Winter wheat	Central NCP			1956-1985	2.0	7.6	185	9.0	1.3	25
Winter wheat	Central NCP			1956-1985	3.0	7.6	185	9.0	1.27	25
Winter wheat	Central NCP			1956-1985	4.0	7.6	185	9.0	1.28	25
Winter wheat	South Henan Province			1956-1985	2.0	8.5	255	8.2	-0.6	25
Winter wheat	South Henan Province			1956-1985	3.0	8.5	255	8.2	-0.57	25
Winter wheat	South Henan Province			1956-1985	4.0	8.5	255	8.2	-0.53	25
Winter wheat	North Anhui and Jiangsu Province			1956-1985	2.0	8.6	301	7.4	0.9	25

Winter wheat	North Anhui and Jiangsu Province			1956-1985	3.0	8.6	301	7.4	0.93	25
Winter wheat	North Anhui and Jiangsu Province			1956-1985	4.0	8.6	301	7.4	0.93	25
Winter wheat	Sanmenxia	34.05	111.03	1992-2009	1.0	8.1	228	11.6	-15.5	26
Winter wheat	Luoyang	34.82	112.43	1992-2009	1.0	9.8	207	8.7	-14	26
Winter wheat	Nanyang	33.03	112.58	1992-2009	1.0	9.8	224	8.6	-4.1	26
Winter wheat	Pingdingshan	34.18	112.83	1992-2009	1.0	9	190	10.0	-2.2	26
Winter wheat	Jiyuan	35.10	112.40	1992-2009	1.0	9.3	199	8.9	2.6	26
Winter wheat	Jiaozuo	35.20	113.20	1992-2009	1.0	10.6	201	8.9	0.0	26
Winter wheat	Zhengzhou	34.72	113.65	1992-2009	1.0	10.2	212	9.2	-4.4	26
Winter wheat	Xuchang	34.01	113.85	1992-2009	1.0	9	199	9.3	2.2	26
Winter wheat	Luohe	33.60	114.00	1992-2009	1.0	9.4	250	8.9	4.8	26
Winter wheat	Zhumadian	33.00	114.01	1992-2009	1.0	9.9	305	8.2	5.5	26
Winter wheat	Xinyang	32.13	114.05	1992-2009	1.0	10.4	408	7.9	2.2	26
Winter wheat	Zhoukou	33.78	114.51	1992-2009	1.0	9.2	220	8.9	8.1	26
Winter wheat	Shangqiu	34.45	115.66	1992-2009	1.0	9.6	234	9.0	2.2	26
Winter wheat	Kaifeng	34.53	114.78	1992-2009	1.0	9.6	239	8.7	4.1	26
Winter wheat	Xinxiang	35.31	113.88	1992-2009	1.0	9.7	158	9.0	2.6	26
Winter wheat	Anyang	36.12	114.40	1992-2009	1.0	9	148	9.5	3.7	26
Winter wheat	Hebi	35.70	114.30	1992-2009	1.0	9.1	174	9.3	1.8	26
Winter wheat	Puyang	35.70	115.01	1992-2009	1.0	8.7	190	9.3	2.9	26
Winter wheat	South China			1975-2008	1.0	9.9	329	6.9	5.35	27
Winter wheat	North China			1975-2008	1.0	9.9	218	8.9	4.04	27

	China	1979-2000	1.0				-4.14	28
	China	1980-2008	1.0				-2.1	29
	China	1980-2008	1.0				-2.9	29
	China	1981-2006	1.0				-3.9	30
	China	1961-2002	1.0				2.35	31
Winter wheat	North China Plain 1	1981-2009	1.0	6.53	182	9.9	9.4	32
Winter wheat	North China Plain 2	1981-2009	1.0	7.8	206	8.7	1.7	32
Winter wheat	Southeast China	1981-2009	1.0	9.7	379	7.0	-3.1	32
Spring wheat	Northwest China	1981-2009	1.0	17.8	91	10.9	-1.3	32
Winter wheat	Northwest China 1	1981-2009	1.0	5.9	77	10.1	0.7	32
Winter wheat	Northwest China 2	1981-2009	1.0	19.3	84	11.4	-6.1	32
Spring wheat	Qinghai	1981-2009	1.0	14.7	176	10.7	6.8	32
Winter wheat	Southwest China	1981-2009	1.0	10.6	272	5.4	-2.3	32
	China	1960-2002	1.0				-1.1	33

Supplementary Table 4. The temperature sensitivity of wheat yield and relevant information from statistical models applied in China.

Supplementary References

1. Tian, Y. *et al*. Warming impacts on winter wheat phenophase and grain yield under field conditions in Yangtze Delta Plain, China. *Field Crop. Res.* **134,** 193–199 (2012).
2. Xiao, G. *et al*. Impact of temperature increase on the yield of winter wheat at low and high altitudes in semiarid northwestern China. *Agric. Water Manage.* **97,** 1360–1364 (2010).
3. Xiao, G. *et al*. Effects of temperature increase on water use and crop yields in a pea–spring wheat–potato rotation. *Agric. Water Manage.* **91,** 86–91 (2007).
4. Tan, K., Fang, S. & Ren, S. Experiment study of winter wheat growth and yield response to climate warming. *Acta Meteor. Sin.* **70,** 902–908 (2012). (in Chinese)
5. Xiao, G. *et al*. The impact of rising temperature on spring wheat production in the Yellow River irrigation region of Ningxia. *Acta Meteor. Sin.* **31,** 6588–6593 (2011). (in Chinese)
6. Ding, L., Cheng, H., Liu, Z. & Ren, W. Experimental warming on the rice-wheat rotation agroecosystem. *Plant Sci. J.* **31,** 49–56 (2013). (in Chinese)
7. Hou, R., Ouyang, Z., Li, Y., Wilson, G. V. & Li, H. Is the change of winter wheat yield under warming caused by shortened reproductive period? *Ecol. evol.* **2,** 2999–3008 (2012).
8. Cao, M. Effects of different nitrogen rates on grain yield and quality formation under temperature increase scenario in wheat and physiology mechanism. *M.S. Thesis. Nanjing Agric. Univ.,* Nanjing. 84 pp (2012). (in Chinese)

9. Liu, L. *et al*. Warming and nitrogen fertilization effects on winter wheat yields in northern China varied between four years. *Field Crop Res.* **151,** 56–64 (2013).
10. Huang, H. Effects of warming and precipitation on spring wheat in the semi-arid region, and response of crop layout on regional climate change. *Ph.D. Thesis, Gansu Agric. Univ.,* Lanzhou. 129 pp (2013). (in Chinese)
11. Zhu, D. & Jin, Z. Impacts of Changes in Both Climate and Its Variability on Food Production in Northeast China. *Acta Agron. Sin.* **34,** 1588–1597 (2008). (in Chinese)
12. Zhou, L., Wang, H. & Zhu, H. Simulation study on the impact of climate warming on production of winter wheat in Huang-Huai-Hai Plain of China. *J. PLA Univ. Sci. Technol.* **4,** 76–82 (2003). (in Chinese)
13. Li, G. *et al.* The effects of climate change on dryland wheat production under different tillage systems. *Acta Prataculturae Sin.* **21,** 160–168 (2012). (in Chinese)
14. Zhang, J., Zhao, Y., Wang, C. & He, Y. Effects of climate change on winter wheat growth and yield in North China. *Chinese J. Appl. Ecol.* **17,** 1179–1184 (2006). (in Chinese)
15. Liu, S. *et al*. Crop yield responses to climate change in the Huang-Huai-Hai Plain of China. *Agric. Water Manage.* **97,** 1195–1209 (2010).
16. Li, S. *et al*. Simulating the impacts of global warming on wheat in China using a large area crop model. *Acta Meteor. Sin.* **24,** 123–135 (2010).
17. Li, G. *et al.* Response of dryland spring wheat yield to elevated CO_2 concentration

and temperature by APSIM model. *Chinese J. Eco-Agric.* **20,** 1088–1095 (2012). (in Chinese)

18. Cong, Z., Wang, S. & Ni, G. Simulations of the impact of climate change on winter wheat production. *J. Tsinghua Univ. (Sci. & Technol.)* **48,** 1426–1430 (2008). (in Chinese)
19. Hao, Q. Simulation of the impacts of climate change on wheat production in the Northwest region. *M.S. Thesis. Beijing For. Univ.*, Beijing. 82 pp (2009). (in Chinese)
20. Yuan, J. Analysis on impact of wheat production and adaptation measures to climate change. *M.S. Thesis. Chinese Acad. Agric. Sci.*, Beijing. 75 pp (2008). (in Chinese)
21. Zhang, X. & Liu, W. Simulating potential response of hydrology, soil erosion, and crop productivity to climate change in Changwu tableland region on the Loess Plateau of China. *Agric. For. Meteorol.* **131,** 127–142 (2005).
22. Xiao, D. & Tao, F. Contributions of cultivars, management and climate change to winter wheat yield in the North China Plain in the past three decades. *Eur. J. Agron.* **52,** 112–122 (2014).
23. Cui, J., Wang, X., Xin, X. & Wu, W. A analysis of the impacts of climate change during crop growing period on yields of major grain crops in China. *Chinese Rural Econ.* (9), 13–22 (2011). (in Chinese)
24. Geng, T., Fu, W. & Chen, C. Response of growth development process and yield of winter wheat to climate warming in Jiangsu province during last 20 Years. *J.*

Triticeae Crops **32,** 1183–1191 (2012). (in Chinese)

25. Wang, S. & Wang, F. A preliminary modelling of effects of climate change on winter wheat yield on the plain of Yellow-Huai-Hai River basins. *Acta Meteor. Sin.* **51,** 209–216 (1993). (in Chinese)
26. Geng, T., Fu, W., Chen, Q., Hou, W. & Chen, C. Spatial-temporal feature of climatic resources and adaptation of winter wheat during last 20 years in Henan province. *J. Triticeae Crops* **33,** 652–661 (2013). (in Chinese)
27. Cui, J., Wang, X. & Xin, X. On the impact of climate change on grain production in China. *Comp. Econ. Soc. Syst.* (2), 54–60 (2011). (in Chinese)
28. You, L., Rosegrant, M. W., Wood, S. & Sun, D. Impact of growing season temperature on wheat productivity in China. *Agric. For. Meteorol.* **149,** 1009–1014 (2009).
29. Zhang, T. & Huang, Y. Estimating the impacts of warming trends on wheat and maize in China from 1980 to 2008 based on county level data. *Int. J. Clim.* **33,** 699–708 (2013).
30. Xiong, W., Holman, I. P., You, L., Yang, J. & Wu, W. Impacts of observed growing-season warming trends since 1980 on crop yields in China. *Reg. Environ. Change* **14,** 7–16 (2014).
31. Lobell, D. B. *et al*. Prioritizing climate change adaptation needs for food security in 2030. *Science* **319,** 607–610 (2008).
32. Tao, F. *et al*. Responses of wheat growth and yield to climate change in different climate zones of China, 1981-2009. *Agric. For. Meteorol.* **91,** 189–190 (2014).

33. Lobell, D. B., Schlenker, W. & Costa-Roberts, J. Climate trends and global crop production since 1980. *Science* **333,** 616–620 (2011).
